# On the Iodide of Potassium in the Latter Stages of Pneumonia

**Published:** 1844-06-29

**Authors:** Geo. L. Upshur

**Affiliations:** Norfolk, Virginia


// THE MEDICAL EXAMINER,

And Record of Medical Science.

Vol. VII.] PHILADELPHIA, SATURDAY, JUNE 29, 1844. [No. 13.

### ON THE IODIDE OF POTASSIUM IN THE LATTER STAGES OF PNEUMONIA.

By Geo. L. Upshur, M. D., of Norfolk, Virginia.

Among the numerous diseased states of the system in which iodide of potassium has been tried, with a success, in many instances, almost miraculous, I have no where seen its use recommended in the suppurative stage of pneumonia. My attention was first directed to it, in this affection, in the early part of last January. A poor woman, about thirty years of age, had suffered from inflammation of the lungs for eleven days before I saw her, without taking any remedy except a dose of castor oil at the commencement, and a dose of sulph. of magnesia on the sixth day. The middle lobe of the right lung was wholly consolidated posteriorly, as was conclusively shown by the bronchial respiration, the crepitant ronchus, and the flat percussion. The patient had suffered considerably, during the past autumn, from intermittent fever, which had left her pale and emaciated. This fact, coupled with the time that had elapsed from the beginning of the disease when I paid my first visit, induced me to forego the use of the more active antiphlogistic remedies. I placed her upon a weak solution of tartar emetic, combined with oxymel of squill. On the third day, after I saw her, and the fourteenth of her attack, the fever was so much lessened, that I ordered a blister posteriorly to the right lung. The cough now became more troublesome, and the sputa were fast losing the *iron-rust* appearance so peculiar to the second stage of pneumonia, and were assuming a character more decidedly purulent.

As the suppurative stage advanced, the patient, as is usual, became more feeble, and, in three or four days from the application of the blister, the collection of pus was so rapid that she scarcely had strength to expectorate it, and was in imminent danger of suffocation on several occasions. The treatment consisted at first of tonics, and stimulating expectorants, which would assist the expectoration for two or three hours only; the system soon losing its susceptibility to their action. It was at this time, when the powers of life were nearly exhausted, that I prescribed the iodide of potassium, in doses of one scruple in twenty-four hours, administered in infusion of hops. The patient commenced to mend after the first day. The expectoration was more easily performed;—the pulse lost its sharp, irritable beat; the appetite improved, and in one week the patient was able to sit up.

Since January, I have had an opportunity to administer this remedy in six other cases of pneumonia, with a detailed account of which it is unnecessary to trespass further upon the columns of the Examiner: suffice it to say, that in no instance did it fail promptly to answer my expectations. It may not be amiss further to state that, as a general rule, the cough, which often harasses the patient for weeks after convalescence, was not so protracted, nor so distressing during its continuance, in the cases where the potassium was used throughout the suppurative stage.

The indications for its use, I have found to be, chiefly, the following: 1. In those cases of pneumonia, arising in anemic persons, where the disease is characterised in its early stages by typhoid symptoms. 2. In cases where inflammatory action, in the commencement, high, has been reduced by antiphlogistic treatment, and the suppurative stage is just beginning. This stage is easily recognised by a sudden depression of the vital powers; by a soft but irritable pulse, and by the bronchial respiration being accompanied by a harsh mucous ronchus. Lastly, in those cases grafted upon long continued intermittents which have left the blood, in a great degree, impoverished.

The *modus operandi* of the iodide of potassium, like that of other alteratives, has never been satisfactorily settled. That it is a medicine of great power, and calculated to do much good in proper hands, no one will pretend to deny. Its action seems to be incompatible with anæmia, and the diseases which result from it;—and with all forms of disease in which the fluids are materially deranged. Doubtless future investigations will throw further light upon its *modus operandi*;—in the interim, it is sufficient for us to be governed in its administration by the light of experience, even at the hazard of being called *empirics*.

*Norfolk*, (*Va.*,) June 16th, 1844.

